# Abcès du sein: aspects épidémiologiques, diagnostiques et thérapeutiques à l’Hôpital Principal de Dakar

**DOI:** 10.11604/pamj.2020.37.16.24694

**Published:** 2020-09-04

**Authors:** Magatte Faye, Birame Ndiaye, Eugène Gaudens Prosper Amaye Diémé, Ibrahima Sall, Samba Thiapato Faye, Oumar Fall, Alamaso Sow

**Affiliations:** 1 Service de Chirurgie Générale, Hôpital Principal de Dakar, Dakar, Sénégal

**Keywords:** Abcès du sein, aspects épidémiologiques, diagnostiques, thérapeutiques, Dakar, Breast abscess, epidemiological features, diagnostic, therapeutic, Dakar

## Abstract

Les abcès du sein sont des collections purulentes néoformées au niveau de la glande mammaire et du tissu péri glandulaire. Ils peuvent être lactants ou non lactants. L’objectif de notre étude était de décrire leurs caractéristiques épidémiologiques, diagnostiques et thérapeutiques au Service de Chirurgie Générale de l’Hôpital Principal de Dakar. Nous avions réalisé une étude rétrospective, descriptive sur une période de 4 ans portant sur tous les patients pris en charge pour un abcès du sein. Nous avions colligé 41 dossiers de patients tous de sexe féminin. L’âge moyen était de 31 ans. Le principal facteur de risque objectivé était la mastite au cours de l’allaitement (27%). Le délai moyen d’évolution était de 12 jours avec des extrêmes de 1 et 30 jours. L’abcès siégeait à gauche dans 61% des cas et se présentait le plus souvent sous la forme d’une tuméfaction inflammatoire (88%). Les quadrants supérieurs étaient le plus souvent concernés (43,9%). L’échographie mammaire était réalisée chez 51,2% des patientes. Le drainage chirurgical sous anesthésie générale était réalisé chez toutes les patientes. La quantité moyenne de pus était de 119 cc. Le germe le plus fréquemment isolé était le Staphylococcus aureus (79,5%). La durée moyenne d’hospitalisation était de 7 jours et la morbidité opératoire de 31,7%. La mortalité était nulle. La prévention des abcès lactants passe par l’enseignement des méthodes d’allaitement et l’antibiothérapie précoce en cas de mastite.

## Abstract (en)

Breast abscesses are neoformed collections of purulent matter occupying the mammary gland and the periglandular tissue. They can occur both in lactating or non-lactating women. The purpose of our study was to describe the epidemiological, diagnostic and therapeutic features of breast abscesses in patients hospitalized in the Division of General Surgery at the main hospital in Dakar. We conducted a retrospective, descriptive study involving all patients treated for breast abscess over a 4-year period. We collected data from 41 medical records of female patients. The average age of patients was 31 years. The main risk factor was mastitis during lactation (27%). The average time of progression was 12 days, ranging from 1 to 30 days. Left abscesses were reported in 61% of cases and mostly manifested as an inflammatory swelling (88%). Upper quadrants were mainly involved (43.9%). Breast ultrasound was performed in 51.2% of female patients. Surgical drainage under general anesthesia was performed in all female patients. The mean quantity of pus was 119 cc. Staphylococcus aureuswas the most commonly isolated germ (79.5%). The average length of stay in hospital was 7 days and operative morbidity was 31.7%. Mortality was zero. Prevention of abscesses during lactation is based on improving breastfeeding practices and on early antibiotic treatment in patients with mastitis.

## Introduction

Les abcès du sein sont des collections purulentes au niveau de la glande mammaire ou du tissu périglandulaire [1]. On distingue les abcès lactants ou puerpéraux (survenant pendant l’allaitement) et les abcès non lactants (non puerpéraux). Le traitement est chirurgical avec une mise à plat de la collection abcédée, mais on observe un essor de la ponction échoguidée [2-4]. L’objectif de notre étude était de décrire leurs caractéristiques épidémiologiques, diagnostiques et thérapeutiques dans notre structure.

## Méthodes

Notre étude était rétrospective, descriptive sur une période de 4 ans (janvier 2014 - décembre 2018), au Service de Chirurgie Générale de l’Hôpital Principal de Dakar. Nous avions inclus tous les patients présentant un abcès de sein diagnostiqué cliniquement ou documenté par l’échographie. Les paramètres étudiés étaient épidémiologiques (âge, sexe, facteurs de risque), cliniques (taille de l’abcès et la localisation), paracliniques (échographie et biologie), thérapeutiques et pronostiques.

## Résultats

**Figure 1:**
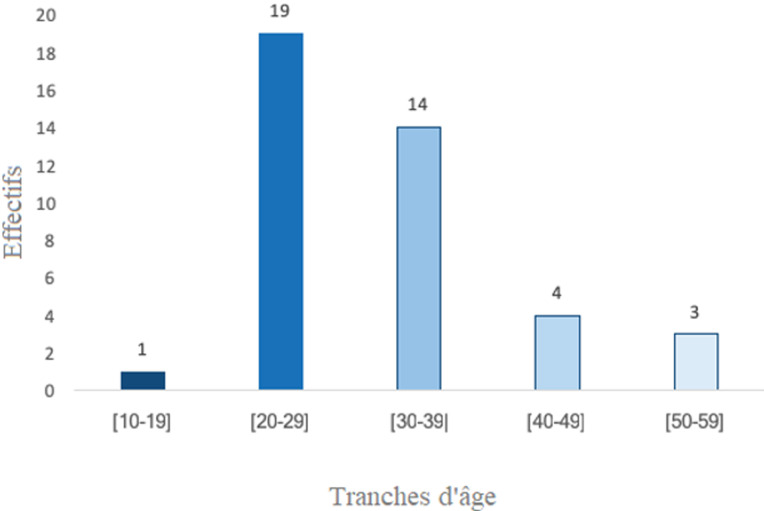
répartition selon l’âge

**Aspects épidémiologiques:** nous avions colligé 41 dossiers de patients tous de sexe féminin. Les abcès représentaient 25% (41/161) de la consultation de sénologie. L’âge moyen des patientes était de 31 ans avec des extrêmes de 13 et 55 ans. La tranche d’âge la plus représentée était celle allant de 20 à 29 ans (Figure 1). Le principal facteur de risque objectivé était la mastite pendant l’allaitement, retrouvée chez 65,9% des patientes (n = 27). Un diabète était retrouvé chez 4,9% des patientes et deux patientes étaient en période de sevrage. Dans 9 cas il n’y avait pas de facteurs de risque rapportés (Tableau 1).

**Tableau 1:** répartition des patients selon le facteur de risque

Facteurs de risque	Effectif	Pourcentage(%)
Nourrice (mastite)	27	65,9
Diabète	2	4,9
Sevrage	2	4,9
Diabète+nourrice	1	2,4
Pas de facteurs	9	21,9
**Total**	41	100

**Figure 2:**
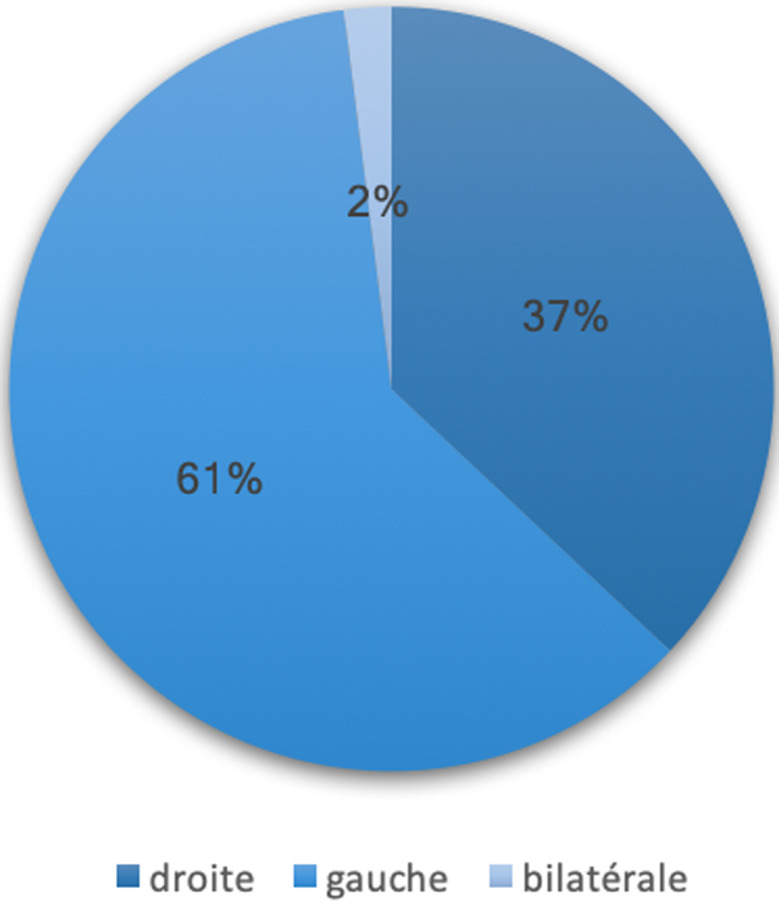
répartition selon la localisation de l’abcès

**Figure 3:**
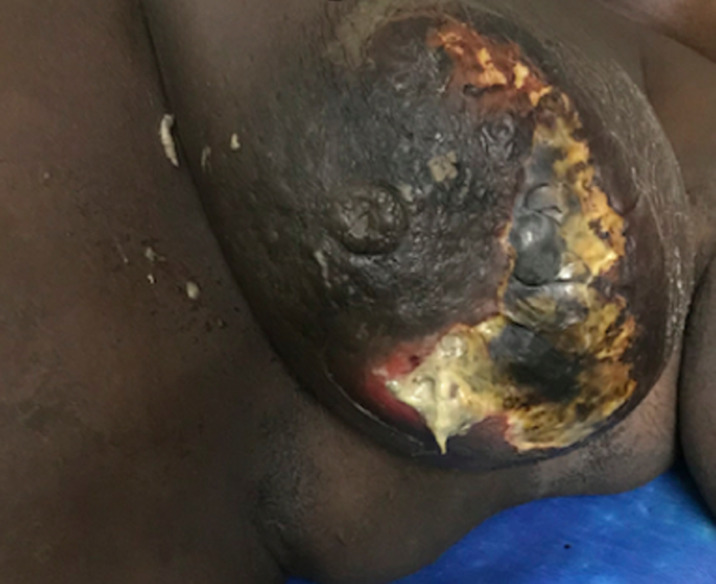
abcès du sein gauche fistulisé

**Aspects diagnostiques:** le délai moyen d’évolution était de 12 jours avec des extrêmes de 1 et 30 jours. L’abcès du sein était localisé à gauche chez 61% des patientes et bilatéral dans 2% des cas (Figure 2). L’abcès se présentait sous la forme d’une tuméfaction inflammatoire dans la majorité des cas (88%), il était déjà fistulisé chez 12% des patientes (Figure 3). Les quadrants supérieurs étaient concernés dans 43,9% des cas (Tableau 2). La taille de l’abcès a été précisée chez 14 patientes, elle était en moyenne de 5,3 cm avec des extrêmes de 3 et 12 cm. L’échographie mammaire était réalisée chez 51,2% des patientes, une patiente avait bénéficié en plus d’une mammographie. Le taux moyen de globules blancs était de 11066/mm^3^.

**Tableau 2:** localisation de l’abcès selon le quadrant

Quadrant	Effectif	Pourcentage(%)
Quadrant supéro externe	8	19,5
Quadrants supérieurs	7	17,1
Quadrant supéro interne	3	7,3
Quadrants externes	5	12,2
Quadrants internes	3	7,3
Quandrant inféro externe	3	7,3
Tous quadrants	3	7,3
Quadrants inférieurs	1	2,5
Non précisé	6	12,2
**Total**	**41**	**100**

**Aspects thérapeutiques et pronostiques:** un drainage chirurgical sous anesthésie générale a été réalisé chez toutes les patientes. Il consistait en une incision puis effondrement des logettes associée à une évacuation du pus et une toilette au sérum salé isotonique. La quantité moyenne de pus était de 119 cc (1-300). Sur les 39 prélèvements de pus qui nous étaient parvenus, le *Staphylococcus aureus* était le germe le plus isolé (79,5%), suivi du Streptocoque non groupable dans 2 cas, puis du *Klebsiella pneumoniae* (1 cas). Cependant, deux cultures étaient stériles (Tableau 3). Une seule patiente âgée de 55 ans et diabétique avait bénéficié d’une biopsie. L’examen anatomopathologique avait objectivé une mastite. Une antibiothérapie à base de ceftriaxone et métronidazole secondairement adaptée à l’antibiogramme a été instituée chez toutes nos patientes pendant une durée de 15 jours. Les pansements se faisaient sous anesthésie générale au bloc opératoire. Un tire lait était prescrit pour les nourrices. La durée moyenne d’hospitalisation était de 7 jours (1-28). La morbidité opératoire était de 31,7%. Les complications notées étaient essentiellement représentées par les suppurations persistantes (grade IIIa de Clavien Dindo), jugulées par des pansements au bloc opératoire et une antibiothérapie adaptée. Une patiente diabétique et nourrice avait présenté un coma acido cétosique nécessitant son admission en réanimation avec une évolution favorable. Aucun décès n’avait été enregistré.

**Tableau 3:** germes isolés dans le pus

Germe	Effectif	Pourcentage(%)
*Staphyloccocus aureus*	31	79,5
Streptocoque non groupable	2	5,1
*Klebsiella pneumoniae*	1	2,6
Stérile	2	5,1
Non contributive	3	7,7
**Total**	**39**	**100**

## Discussion

**Épidémiologie: incidence et facteurs de risques:** les abcès du sein peuvent représenter jusqu'à 3% des références dans les centres de sénologie [5]. Dans notre étude, l’incidence est plus élevée; 25% de la consultation de sénologie. Celle de l’abcès lactant varie de 0,1 à 0,52% dans la littérature [4, 6-8]. Dans les séries occidentales, ces taux bas s’expliquent par la prescription précoce d’une antibiothérapie en cas de mastite [9], tandis que dans notre étude les patientes sont vues tardivement avec un délai moyen de consultation de 12 jours. Les abcès sont plus fréquemment diagnostiqués chez les femmes allaitantes [10]. Dans notre étude 68,2% des abcès étaient lactants. La mastite est le principal facteur de risque de l’abcès lactant. Debord *et al*. ont rapporté un taux de mastite de 91,1% [4]. Elle est souvent secondaire à une stase lactée favorisée par une difficulté de mise au sein dès la maternité (douleur, crevasse, frein de langue, difficulté de succion du nouveau-né), par une reprise du travail, par un sevrage ou par des positions d’allaitement inchangées [4]. C’est davantage la stase lactée secondaire à la diminution des tétées en raison de la douleur qui est responsable de l’abcès que la crevasse elle-même comme porte d’entrée bactérienne [11]. Les abcès non puerpéraux sont favorisés par une anomalie anatomique (inversion du mamelon, ectasie des canaux galactophores, métaplasie malpighienne des sinus lactifères ou piercing) ou par des facteurs généraux (diabète, obésité, immunodépression). L’homme peut être touché, sans âge de prédilection [1,12]. Le tabagisme peut également être un facteur de risque pour les abcès non lactants [13]. Parmi nos patientes présentant des abcès non lactants (n = 13), 4 étaient diabétiques et pour les 9 autres patientes, il n’y avait pas de facteur de risque identifié.

**Aspects cliniques:** les signes cliniques associés le plus souvent la douleur, la rougeur et la tuméfaction [1,9]. Les signes inflammatoires étaient présents chez 88% de nos patientes, et 12% des patientes avaient déjà un abcès fistulisé. Les abcès puerpéraux sont généralement profonds ou centromammaires, tandis que les abcès non puerpéraux sont souvent superficiels et para aréolaires [1]. La localisation profonde ou superficielle n’a pas été précisée dans les dossiers de nos patientes. L’abcès du sein est généralement unilatéral et touche dans les mêmes proportions le sein droit et le sein gauche [1, 14, 15]. Cependant dans notre étude, la localisation gauche était prédominante (61%), avec un cas d’abcès bilatéral chez une nourrice diabétique. L’abcès se localise le plus souvent dans la partie supérieure du sein; 43,9% chez nos patientes dont 19,5% pour la partie supéro externe. Ceci s’explique par le fait que le quadrant supéro externe est plus riche en parenchyme glandulaire et que la partie supérieure du sein est moins bien drainée étant donnée la position d’allaitement du bébé souvent identique [4].

**Paraclinique:** l’échographie est l’examen de choix pour confirmer le diagnostic [1, 10, 16]. La mammographie a un rôle limité dans le diagnostic d’abcès du sein; elle est réalisée si la symptomatologie ne cède pas malgré le traitement afin d’éliminer un cancer inflammatoire [1]. La fréquence d’une association entre un abcès et un cancer varie de 2 à 20% [14, 16-18]. Dans notre étude, l’échographie mammaire a été réalisée dans 51,2% des cas, une patiente était reçue avec une mammographie déjà réalisée en externe et qui n’était pas contributive.

**Bactériologie:** le *Staphylococcus aureus* était le germe le plus noté dans notre série (79,5%). Cette prédominance a également été retrouvée dans la littérature. En effet c’est le germe aérobie le plus fréquemment isolé dans les abcès puerpéraux [11, 19] et non puerpéraux avec l’émergence de Staphylocoque aureus méticilline-résistant (SARM) [20]. Cependant, chez deux de nos patientes la culture était stérile. Une culture stérile peut correspondre aux faux négatifs dus à une précédente antibiothérapie, mais aussi à des germes nécessitant des cultures spécialisées (en particulier les mycobactéries et certaines bactéries anaérobies) [20]. Le Peptostreptocoque et le *Propionibacterium* sont des germes anaérobies isolés dans les infections aiguës, chroniques ou récidivantes; on les trouve souvent associés à d’autres germes anaérobies ou aérobies [9,21,22].

**Traitement:** notre attitude thérapeutique consistait en un drainage chirurgical associée à une antibiothérapie anti staphylococcique secondairement adaptée à l’antibiogramme. Les pansements étaient quotidiens au bloc opératoire, expliquant la durée moyenne d’hospitalisation longue (7 jours). Cependant, la ponction aspiration échoguidée associée à une antibiothérapie est le traitement de première intention de l’abcès du sein [1, 4, 9]. La méthode de Hook qui consiste en une ponction aspiration avec irrigation par une solution isotonique est indiquée si la taille de l’abcès est inférieure à 3 cm. Les aspirations sont répétées si nécessaire [17]. Le taux de succès est de 82 à 85% [3,16]. Si la taille de l’abcès est supérieure à 3 cm, le drainage percutané par un cathéter ou l’irrigation par un antibiotique à large spectre (méthode impériale) est indiquée [1, 3, 18]. Le drainage par mammotome a été décrit par Zhu Quan-Li *et al*. [23]. La ponction présente de nombreux avantages: cicatrice moins disgracieuse, pas d’anesthésie générale, douleur et coût moindres, possibilité de suivi en ambulatoire, pas de séparation mère-enfant. La seule contre-indication de la ponction est le refus de la patiente et l’impossibilité d’une surveillance rapprochée [1]. La ponction aspiration échoguidée n’a pas pu être réalisée chez nos patientes du fait d’une absence d’un plateau technique adéquat. Le drainage chirurgical est indiqué en cas d’échec du traitement percutané [14, 17, 18]. Il consiste à inciser le sein sous anesthésie générale en suivant les lignes de la peau, à effondrer les logettes au doigt, à prélever et à évacuer le pus [9, 24]. Le drainage de la loge par un drain souple n’est pas systématique [9]. Une biopsie des berges doit être réalisée systématiquement en cas d’abcès non puerpéral et chaque fois que les berges sont suspectes en cas d’abcès puerpéral [1]. Dans notre étude une biopsie avait été réalisée chez une patiente de 55 ans diabétique et l’examen anatomopathologique avait objectivé une mastite. L’antibiothérapie peut durer 7 à 14 jours [20]. Pour la poursuite de l’allaitement maternel, les attitudes sont différentes. Le drainage chirurgical impose souvent l’arrêt de l’allaitement du fait de la séparation mère enfant lors de l’hospitalisation [1]. Cependant l’arrêt brutal de l’allaitement majore la stase lactée et peut augmenter la taille de l’abcès voire favoriser sa non-résorption malgré un traitement adéquat [25]. Plusieurs auteurs encouragent la poursuite de l’allaitement maternel [19,26,27], même du côté atteint [24,28], surtout si une ponction a été réalisée [29]. Un tire lait peut être utilisé jusqu’à ce que l’abcès se résorbe [25]. Dans notre étude, un tire lait était prescrit de façon systématique pour diminuer l’engorgement mammaire.

## Conclusion

Les abcès du sein ne sont pas rares sous nos contrées. La prise en charge thérapeutique doit s’orienter vers des moyens moins invasifs (traitement percutané). Il faudra avoir la hantise d’un cancer du sein devant un abcès non puerpéral. La prévention des abcès puerpéraux passera par un meilleur apprentissage des méthodes d’allaitement.

### Etat des connaissances sur le sujet

- Les abcès du sein sont lactants ou non lactants;
- Les abcès du sein sont rares en Occident du fait du traitement précoce de la mastite;
- La ponction écho guidée est le traitement de première intention.

### Contribution de notre étude à la connaissance

- Les abcès du sein sont fréquents en Afrique subsaharienne avec un délai de consultation long;
- Le Staphylococcus aureus est le germe le plus isolé;
- Le traitement chirurgical est une alternative à la ponction échoguidée dans les pays en développement.

## References

[1] Beyrouti MI, Boujelben S, Beyrouti R, Ben Amar M, Abid M, Louati D (2007). Abcès pyogéniques du sein: aspects cliniques et thérapeutiques. Gynecol Obstet Fertil.

[2] Dixon JM (1988). Repeated aspiration of breast abscesses in lactating women. BMJ.

[3] Schwarz RJ, Shrestha R (2001). Needle aspiration of breast abscesses. Am J Surg.

[4] Debord MP, Poirier E, Delgado H, Charlot M, Colin C, Raudrant D (2016). Abcès du sein lactant et si on ne les opérait plus. J Gynecol Obstet Biol Reprod.

[5] McFarlane ME (2001). Benign breast diseases in the Afro Carribean population. East Afr Med J.

[6] Linda Kvist, Hakan Rydhstroem (2005). Factors related to breast abscess after delivery: a population-based study. BJOG.

[7] Branch-Elliman W, Golen TH, Gold HS, Yassa DS, Baldini LM, Wright SB (2012). Risk factors for *Staphylococcus aureus* postpartum breast abscess. Clin Infect Dis.

[8] Lisa Amir H, Della Forster, Helen McLachlan, Judith Lumley (2004). Incidence of breast abscess in lactating women: report from an Australian cohort. BJOG.

[9] Delaloye JF, Brugger CR, Treboux AI, Anaye A, Meuwly JY (2010). Abcès du sein: privilégier la ponction aspiration échoguidée. Rev Med Suisse.

[10] Faisal Elagili, Norlia Abdullah, Lie0077 Fong, Tan Pei (2007). Aspiration of breast abscess under ultrasound guidance: outcome obtained and factors affecting success. Asian J Surg.

[11] Dixon JM (2013). Breast infection. BMJ.

[12] Friedolf Peters, Anja Kiesslich, Volker Pahnke (2002). Coincidence of non-puerperal mastitis and non-inflammatory breast cancer. Eur J Obstet Gynecol Reprod Biol.

[13] O’Brien C, Quinn E, Murphy M, Lehane E, O’Leary DP, Livingstone V (2020). Breast abscess: not just a puerperal problem. Breast J.

[14] Juan Berna-Serna D, Manuel Madrigal, Juan Berna-Serna D (2004). Percutaneous management of breast abscesses: an experience of 39 cases. Ultrasound Med Biol.

[15] Karstrup S, Solvig J, Nolsøe CP, Nilsson P, Khattar S, Loren I (1993). Ultrasonically guided percutaneous drainage of breast abscesses. Radiology.

[16] O’Hara RJ, Dexter SP, Fox JN (1996). Conservative management of infective mastitis and breast abscesses after ultrasonographic assessment. Br J Surg.

[17] Hook GW, Ikeda DM (1999). Treatment of breast abscesses with US-guided percutaneous needle drainage without indwelling catheter placement. Radiology.

[18] Imperiale A, Zandrino F, Calabrese M, Parodi G, Massa T (2001). US-guided serial percutaneous and local antibiotic therapy after unsuccessful systemic antibiotic therapy. Acta Radiol.

[19] Ulitzsch D, Nyman MKG, Carlson RA (2004). Breast abscess in lactating women: US-guided treatment. Radiology.

[20] Laas E, Touboul C, Kerdraon O, Catteau-Jonard S (2015). Mastites inflammatoires et infectieuses du sein en dehors de la grossesse et de la période d’allaitement: recommandations. J Gynecol Obstet Biol Reprod (Paris).

[21] Edmiston CE Jr, Walker AP, Krepel CJ, Gohr C (1990). The non-puerperal breast infection: aerobic and anaerobic microbial recovery from acute and chronic disease. J Infect Dis.

[22] Itzhak Brook (1988). Microbiology of non-puerperal breast abscesses. J Infect Dis.

[23] Zhu Quan-Li (2020). Clinical study of mammary abscess treatment during lactation by using Mammotome rotatory puncture and drainage. Breast J.

[24] Dixon JM (1992). Outpatient treatment of non-lactational breast abscesses. Br J Surg.

[25] Leila Cusack, Meagan Brennan (2011). Lactational mastitis and breast abscess: diagnosis and management in general practice. Aust Fam Physician.

[26] Eryilmaz R, Sahin M, Hakan Tekalioglu M, Daldal E (2005). Management of lactational breast abscesses. Breast.

[27] Sarhan HH, Ibraheem OM (2012). Percutaneous needle aspiration is a minimally invasive method for a breast abscess. Arch Clin Exp Surg.

[28] Tewari M, Shukla HS (2006). An effective method of drainage of puerperal breast abscess by percutaneous placement of suction drain. Indian J Surg.

[29] Pamela Berens, Laurie Swaim, Bethany Peterson (2010). Incidence of methicillin resistant *Staphylococcus aureus* in postpartum breast abscesses. Breastfeed Med.

